# Efficacy, Use, and Acceptability of a Web-Based Self-management Intervention Designed to Maximize Sexual Well-being in Men Living With Prostate Cancer: Single-Arm Experimental Study

**DOI:** 10.2196/21502

**Published:** 2021-07-26

**Authors:** Sean R O'Connor, Carrie Flannagan, Kader Parahoo, Mary Steele, Samantha Thompson, Suneil Jain, Michael Kirby, Nuala Brady, Roma Maguire, John Connaghan, Eilis M McCaughan

**Affiliations:** 1 Centre for Public Health Queen's University Belfast Belfast United Kingdom; 2 Institute of Nursing & Health Research Ulster University Newtownabbey United Kingdom; 3 Centre for Clinical and Community Applications of Health Psychology Faculty of Social and Human Sciences University of Southampton Southampton United Kingdom; 4 Urology Department Belfast City Hospital Belfast United Kingdom; 5 Clinical Oncology Northern Ireland Cancer Centre Belfast United Kingdom; 6 Faculty of Health and Human Sciences University of Hertfordshire Hatfield United Kingdom; 7 The Prostate Centre London United Kingdom; 8 Northern Health and Social Care Trust Antrim United Kingdom; 9 Department of Computer and Information Sciences University of Strathclyde Glasgow United Kingdom

**Keywords:** prostate cancer, sexual well-being, digital interventions, self-management

## Abstract

**Background:**

Sexual dysfunction is a frequent side effect associated with different prostate cancer treatment approaches. It can have a substantial impact on men and their partners and is associated with increased psychological morbidity. Despite this, sexual concerns are often not adequately addressed in routine practice. Evidence-based web-based interventions have the potential to provide ongoing information and sexual well-being support throughout all stages of care.

**Objective:**

The aim of this study is to examine the efficacy of a web-based self-management intervention designed to maximize sexual well-being in men living with prostate cancer and explore user perspectives on usability and acceptability.

**Methods:**

We used a single-arm study design, and participants were provided with access to the 5-step intervention for a period of 3 months. The intervention content was tailored based on responses to brief screening questions on treatment type, relationship status, and sexual orientation. Efficacy was assessed by using two-tailed, paired sample *t* tests for comparing the mean differences between pre- and postintervention measurements for exploring the participants’ self-reported knowledge and understanding, sexual satisfaction, and comfort in discussing sexual issues. Usability and acceptability were determined based on the program use data and a postintervention survey for exploring perceived usefulness.

**Results:**

A total of 109 participants were recruited for this study. Significant postintervention improvements at follow-up were observed in the total scores (out of 20) from the survey (mean 12.23/20 points, SD 2.46 vs mean 13.62/20, SD 2.31; *t*_88_=9.570; *P*=.001) as well as in individual item scores on the extent to which the participants agreed that they had sufficient information to manage the impact that prostate cancer had on their sex life (mean 2.31/4 points, SD 0.86 vs mean 2.57/4, SD 0.85; *t*_88_=3.660; *P*=.001) and had the potential to have a satisfying sex life following treatment (mean 2.38/4 points, SD 0.79 vs mean 3.17/4, SD 0.78; *t*_88_=7.643; *P*=.001). The median number of intervention sessions was 3 (range 1-11), and intervention sessions had a median duration of 22 minutes (range 8-77). Acceptable usability scores were reported, with the highest result observed for the question on the extent to which the intervention provided relevant information.

**Conclusions:**

This study provides evidence on the efficacy of a tailored web-based intervention for maximizing sexual well-being in men living with prostate cancer. The results indicate that the intervention may improve one’s self-perceived knowledge and understanding of how to manage sexual issues and increase self-efficacy or the belief that a satisfactory sex life could be achieved following treatment. The findings will be used to refine the intervention content before testing as part of a larger longitudinal study for examining its effectiveness.

## Introduction

### Background

Prostate cancer is the most common cancer among men, accounting for approximately 25% of all cases [1,2]. Although incidence rates are rising, partly because of improved screening and changes in the population age profile [3], 5- and 10-year survival rates continue to improve [4]. Consequently, an increasing number of men are living with significant long-term side effects associated with different treatment approaches [3]. Sexual challenges are the most frequently occurring sequelae [5,6]. Rates of sexual dysfunction having a moderate to severe impact on quality of life of 31%-64% of the men have been reported after radical prostatectomy and external beam radiotherapy [7,8]. In a recent large-scale survey, 81% of the men reported poor sexual function after treatment, with approximately 56% not being offered any intervention to help manage these concerns [9]. Changes in sexual function are subsequently regarded as a major issue that can result in increased psychological morbidity, including depression and relational dissatisfaction, and reductions in self-efficacy and overall quality of life [10]. Sexual well-being can be described as a complex and highly individualized issue that encapsulates all aspects of sexuality, including physical, emotional, mental, and social aspects [11,12]. Patients and their partners often have complex sexual health and well-being needs following diagnosis and treatment [13,14]. Effective evidence-based care and support are therefore required to help manage these needs. Care and support aimed at maximizing sexual well-being should not be restricted to purely biomedical approaches that focus on erectile dysfunction and physiologic penile rehabilitation [15]. These approaches do not address sexual well-being after prostate cancer diagnosis in a biopsychosocial context [16].

Although there is evidence examining relatively intensive couple-based counseling interventions delivered by health professionals [17,18], there is often limited access to such services. Current treatment guidelines [19,20] endorse the delivery of psychosexual care for patients living with prostate cancer with recommendations made for the minimal level of support that should be provided. This includes provision of individualized information tailored to the patients’ needs and clear advice about the potential long-term side effects of treatment as well as ensuring ongoing access to specialist care, including erectile dysfunction clinics. Despite these recommendations, the information provided varies greatly and is not routinely available across services [9]. Patients and their partners frequently report that they do not receive adequate support to manage these concerns [21,22]. In a study of prostate cancer follow-up at urology and radiotherapy clinics, the sexual aspects of recovery were not discussed in 46% and 48%, respectively, of the observed consultations [23]. The participants’ partners were present in approximately half of the consultations, but their involvement was minimal, and they did not seem to influence whether any discussion of sexual concerns took place [23].

Discussing sexual health concerns in routine practice can be challenging, and there are a number of barriers to engaging in these conversations [24,25]. Health care providers often feel unequipped to deal with sexual health issues and report a lack of resources to offer patients and their partners if they do identify a problem [26]. Patients may not spontaneously report sexual health issues and prefer that health professionals initiate the discussion [27]. These assumptions may be compounded when health professionals work with patients from minority groups such as men who have sex with men. For example, many gay men report that health professionals often fail to ask about sexual orientation during the initial consultations and assume that they are heterosexual [28].

Web-based interventions provide access to ongoing, easily accessible, and adaptable information and support to users at all stages of care [29]. There is evidence that the tailoring or the personalization of web-based information and support interventions is more effective and results in increased user engagement when compared with standardized information [30]. In addition, tailored self-management interventions are more capable of altering determinants of individual beliefs and behaviors [31]. However, some barriers exist that can limit engagement with web-based resources, including a lack of time and usability issues [32]. Despite this, web-based interventions that specify and acknowledge the impact of treatment on the sexual well-being of both men and their partners and provide appropriate support have the potential to improve patient-important outcomes, including sexual well-being satisfaction and quality of life. Such interventions, which are aimed at supporting men and their partners to cope with changes in sexual health and well-being after prostate cancer treatment, require further investigation.

This paper presents an evaluation of a web-based self-management intervention designed to maximize sexual well-being in men living with prostate cancer. The program provides tailored information and support based on the user’s treatment type, relationship status, and sexual orientation. This aligns with existing guidelines that advocate tailored psychosexual interventions [19,20]. It is also in line with recommendations that emphasize early support, consisting of educational approaches and interventions to manage sexual side effects of treatment and minimize the impact of changes to sexual function on the men and their partners [33].

### Objectives

Recent frameworks for developing and evaluating complex health care interventions emphasize a requirement for greater focus on initial development because many fail to demonstrate effectiveness in real-world contexts [34]. Before conducting larger studies exploring intervention effectiveness, this study was conducted to examine if the intervention had any effect on patient-important outcomes and to explore its acceptance to users. Therefore, the primary objective of this study is to examine the efficacy of the intervention in terms of its impact on participants’ understanding of how to manage sexual concerns, comfort in discussing such issues with partners and health professionals, and overall satisfaction with their sex life. The secondary objective is to explore program use and user perspectives on usability and acceptability.

## Methods

### Study Design

A single-arm pilot study design with pre- and postintervention outcome assessments was used. Following enrollment, the participants were given access to the intervention for a 3-month period. Where appropriate, the design and conduct of the study followed the Consolidated Standards of Reporting Trials 2010 statement: extension to randomized pilot and feasibility trials [35].

### Study Setting and Participants

The primary study recruitment methods were through health professionals signposting to the study website men who were attending routine prostate cancer appointments at 2 clinical sites (Northern Ireland Cancer Centre, Belfast City Hospital, Belfast, United Kingdom, and Ninewells Hospital, Dundee, United Kingdom) and through posters and leaflets placed in clinical areas within the same sites. In addition, a link to the program was included in the patient information section of a national prostate cancer charity website. A minimum sample of 81 participants was determined based on two-tailed, paired sample *t* tests, α=.05, and a medium estimated effect size of 0.03 [36]. Therefore, a planned sample size of 100 participants was selected to allow for potential loss of data at follow-up. Following web-based registration on the site, potential participants were required to complete a screening questionnaire before a baseline assessment. To meet the study inclusion criteria, participants were required to be adult males (aged 18 years or older); diagnosed with prostate cancer; and due to start, or be currently receiving, supportive care after radical prostatectomy, external beam radiotherapy, brachytherapy, or androgen deprivation therapy (either alone or in combination). The exclusion criteria were as follows: being on active surveillance or not being able to understand instructions written in English.

### Study Procedures

Ethical approval for the study was provided by the Office for Research Ethics Committees Northern Ireland (reference number: 17/NI/014). Before completing the web-based screening questionnaire, the participants were provided with a study information sheet detailing the nature and purpose of the study. They were also given the opportunity to contact a member of the research team to ask any questions they might have about the study. All participants provided informed consent before participation. Subsequently, they completed the baseline assessment, which included demographic information and baseline outcomes, and provided responses to the 3 questions that were used to enable the intervention to provide tailored information and support based on the responses given (Textbox 1). The participants were given access to the program for 3 months. The only contact that the participants received during the intervention period was through automated emails sent to confirm successful enrollment and to remind them that they had 1 week left to use the intervention. After the 3-month intervention period ended, the participants received email reminders asking them to log in to the website and complete a follow-up assessment in which the baseline outcomes were repeated. They were also asked to complete a questionnaire on program usability and acceptance.

**Questions asked at baseline to allow tailored information to be provided by the intervention.**

**Tailoring questions**

- What treatment have you had?
  Surgery
  Combined radiotherapy and hormone therapy
  Radiotherapy
  Hormone therapy
- Are your sexual partners usually male or female?
  Female
  Male
- Do you currently have a partner?
  Yes
  No

### Intervention Development, Theory, and Description

#### Intervention Development

A systematic, iterative, and theory-based process modeled on the person-based approach was used to inform the development, design, and testing [37]. This method was primarily used to ensure that the development was in close collaboration with end users and to optimize intervention acceptability, feasibility, and engagement. This process included 2 phases: an intervention development and testing phase and an evaluation and follow-up phase. The draft intervention content was modeled on an existing sexual well-being intervention [38]. In the first phase, evidence reviews and a qualitative synthesis of data from semistructured interviews and focus group discussions with end users and field content experts were used to identify the core or essential elements of the intervention. Additional interviews with both types of participants were then used to review and revise the paper-based versions of the content. This was to ensure that it was relevant and meaningful to users. An initial prototype version of the intervention was subsequently built using LifeGuide software (University of Southampton) [39], which provides tools for developers to author, edit, deploy, and trial interventions. Further modifications were made based on usability testing and additional rounds of qualitative interviews. These steps were carried out before making further revisions and building the final version of the intervention that was used for this evaluation. In the second phase, evaluation of the intervention was conducted based on quantitative and qualitative data exploring preliminary efficacy, use, and acceptability data, which are presented in this paper.

#### Theoretical Underpinning

As the intervention was delivered in a web-based format, its theoretical underpinning was based on the unified theory of acceptance and use of technology, a widely used model of technology acceptance and use intention [40]. This model integrates a number of relevant technology acceptance and behavior change theories, including self-efficacy, the theory of reasoned action, technology acceptance theory, the theory of planned behavior, and social cognitive theory. Critical to the theory of acceptance and use of technology model are the concepts of perceived usefulness and ease of use. The central determinants of intention and use are performance and effort expectancy, social influences, and facilitating conditions, with factors such as age, gender, prior experience, and voluntariness to use assumed to be moderators of these effects [41].

#### Intervention Description

The final version of the intervention consisted of a 5-step program designed to maximize sexual well-being in men living with prostate cancer. The 5 steps were as follows: (1) sexual well-being and prostate cancer, (2) changes and coping with changes, (3) maintaining and improving your sex life, (4) exploring sexual pleasure, and (5) facing the future. In addition, a user toolkit containing a series of quick guides was included. Each step varied in length from 12 to approximately 40 webpages. Information was also layered using page tabs, meaning that although all participants were required to view core information, other information could be skipped or viewed at a later date. The intervention provided tailored information and support based on the user’s treatment type, relationship status, and sexual orientation, with the program allowing different information to appear on screen based on the responses given to the brief tailoring questions that the participants completed during initial registration. The participants were encouraged to use the program with their partner, and specific tailored information was included for partners. This included, for example, information for female partners on women’s health and couple communication activities as well as advice on talking to a partner’s health care team. It was recommended that the users complete the steps in sequence over the 3-month intervention period. However, a key design feature of the intervention was that all steps were accessible from the start of the intervention period. It was also emphasized to the participants that the intervention was designed as a resource that they could return to at any time to revisit previously viewed sections or to view or complete unfinished steps. The participants received a tick mark over each step, which could be seen each time they logged in. This was to indicate the steps that they had already completed.

**Figure 1:**
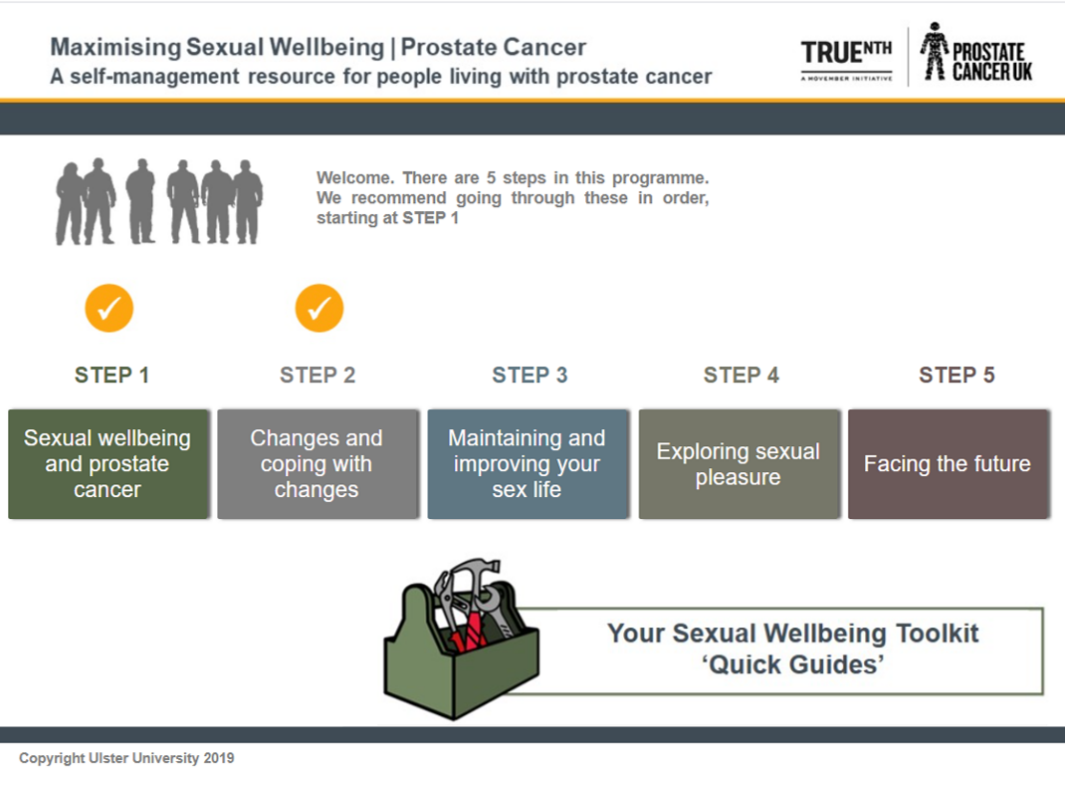
Intervention screenshot.

Each step consisted of a series of webpages containing text-based information, infographics, videos highlighting patient experiences, and instructional videos delivered by health care professionals. Some steps included exercises, activities, and other resources for participants. These included a couple communication activity and a printed checklist for the participants to use when discussing sexual issues or concerns with their health care professional. The intervention content also included important behavior change components and techniques, including use of social support; information about health consequences; instruction on how to perform a behavior; demonstration or modeling of behavior; and use of prompts, reminders, and cues [42]. The key principles and characteristics of the final intervention version are listed in Textbox 2. A screenshot of the intervention home page is shown in Figure 1.

**Key principles and characteristics of the final intervention.**

**Key principles**

- To normalize sexual concerns associated with prostate cancer and its treatment and address patient and partner expectations of potential sexual recovery
  Provide case-based examples, including patient experience videos
  Provide potential side effects information, including common methods of coping and managing individual side effects
- To acknowledge changes or potential loss of sexual function and promote resilience and effective coping strategies
  Promote benefits of adapting to a new sexual normal and adopting new approaches and working as a couple
  Provide instructional and demonstration videos presented by health professionals
- To provide personalized information and support based on needs, including treatment type, sexual orientation, and partner status
  Provide layering of information and support based on needs (ensuring that the intervention can be used for brief periods but can also facilitate more in-depth or intensive support based on user needs)
- To promote increased sexual well-being conversations between partners and health professionals
  Provide printable health professional communication aid
  Provide printable couple communication exercise
  Provide specific supportive information for partners to promote shared intervention use
- To provide usable, easily accessible, and relevant support available at all stages of care
  Include printable exercises and activities to be used as prompts or reminders of key points
  Provide information on appropriate support services based on needs
  Use simple design interface with core information provided on main webpages and selected additional information available based on user preference

### Outcomes

As the objectives of this study are to explore the efficacy of the intervention and examine use data and user perspectives on usability and acceptability, a 3-month pilot study was conducted. Efficacy was assessed using pre- and postintervention measurements of a self-reported web-based survey that included 1 question exploring knowledge and understanding, 2 questions on sexual satisfaction, and 2 questions on comfort in discussing sexual issues (with health care professionals and with a partner). The participants were asked to rate their level of agreement with the 5 different questions using a 4-point Likert scale anchored by *strongly disagree* and *strongly agree* at either end. The composite efficacy score out of 20 was calculated by combining the scores for all 5 questions.

Intervention use was determined by calculating the number of intervention sessions (log-ins) for each participant, the duration of each session, and the total time spent using the intervention over the 3-month evaluation phase. In addition, the participants were classified as *completers* or *noncompleters* based on whether they had completed at least 4 of the 5 intervention steps.

Usability and acceptability were determined based on different methods, including a brief web-based survey and free-text responses to 2 questions asked at the 3-month follow-up assessment. This survey was based on a modified and shortened version of the system usability scale [43]. Modifications were made to ensure that the questions were relevant to the assessment of intervention acceptability. This included the addition of questions on the look and design of the program and the relevance of the information provided. The participants were asked to rate their level of agreement with each of the 6 questions using a 4-point Likert scale anchored by *strongly disagree* and *strongly agree* at either end. A composite usability score out of 24 was calculated by combining the scores for all 6 questions. The participants were also asked whether they would recommend the intervention to others (yes, not sure, or no). Finally, the participants were asked to provide free-text responses to the following questions:

1. Did you gain anything from using the intervention?
2. Do you have any recommendations on how the intervention can be improved?

### Data Analysis

Data were exported into SPSS version 25.0 (IBM Corporation), which was used to provide a descriptive analysis of demographic data, intervention use data, and usability ratings. To assess intervention efficacy, paired sample *t* tests were used to compare the mean pre- and postintervention efficacy measures for the composite and individual question scores. Data were tested for normality of distribution, and a Bonferroni-adjusted *P*=.007 (*P* value of .05 divided by the number of comparisons: n=6) was used to allow for multiple comparisons. Estimated effect sizes for pre-post intervention effects were also calculated using the following criteria: 0.00-0.19, insignificant; 0.20-0.49, small; 0.50-0.79, medium; and ≥0.80, large [44]. Independent sample *t* tests were then used to test for any significant differences between the composite and individual usability question scores between the participants classified as *completers* (those who accessed at least 4 of the 5 intervention steps) and those classified as *noncompleters*.

## Results

### Participant Flow and Retention

**Figure 2:**
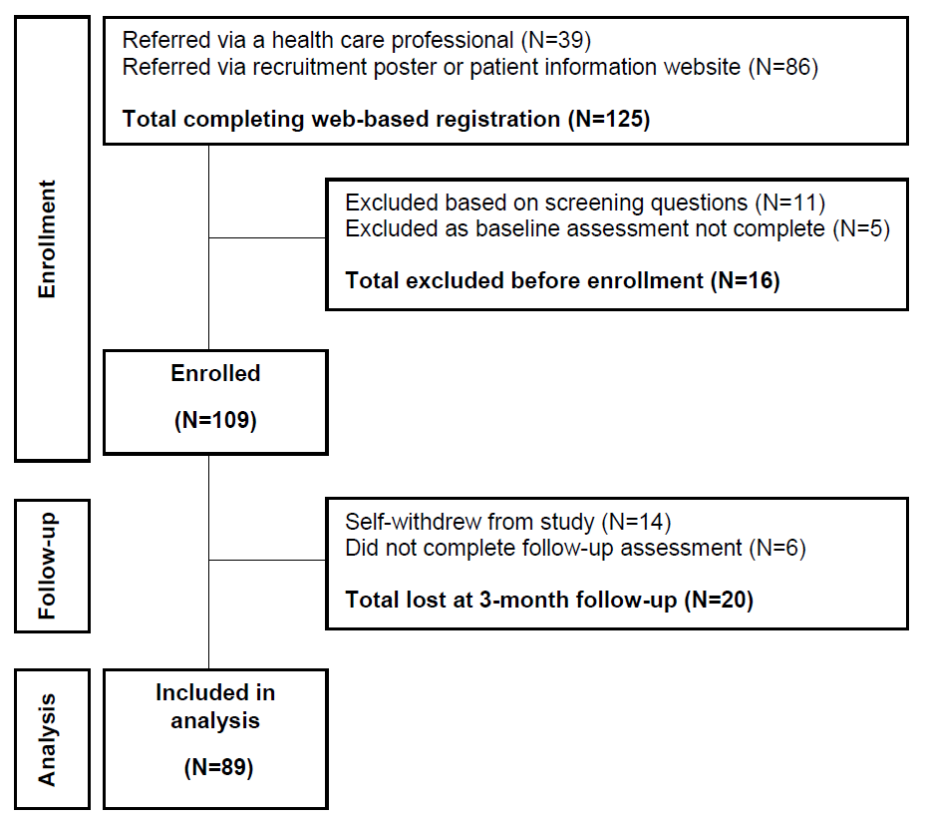
Participant flow diagram.

Participants were recruited for the study between February 2019 and July 2019. A total of 125 potential participants completed the initial web-based registration; however, 16 of these participants were not enrolled on the basis of their responses to the screening questions or because they did not complete the baseline assessment questions. Therefore, a total of 109 men were enrolled in the study, and they provided informed consent to participate (Figure 2). Of the 109 men, 20 (18.3%) were lost to follow-up at 3 months owing to self-withdrawal (defined as no intervention use or log-ins after initial registration) or noncompletion of follow-up assessment questionnaires; this resulted in data from 89 (81.7%) participants being included in the final analysis.

### Participant Demographics

Most men (66/89, 74%) were aged between 50 and 69 years, and most of them (83/89, 93%) were from a White ethnic background. Surgical intervention alone (not in combination with any other form of treatment) was the single most common form of treatment received (53/89, 60%), with combined treatment (including radiotherapy and hormone therapy) being the second most common form of treatment (20/89, 22%). Full demographic details of the participants are shown in Table 1. There were no observable differences in demographics between the 89 men included in the analysis and the 20 men who were lost to follow-up.

**Table 1:** Demographic details of participants enrolled in the study (N=109).

Demographics			Included in analysis (n=89), n (%)		Withdrawals (n=20), n (%)
**Age category (years)**					
	18-49	1 (1)		0 (0)	
	50-69	66 (74)		16 (80)	
	≥70	22 (25)		4 (20)	
**Ethnicity**					
	White	83 (93)		19 (95)	
	Asian or Asian British	1 (1)		0 (0)	
	Black, African, Caribbean, or Black British	4 (5)		1 (5)	
	Other	1 (1)		0 (0)	
**Previous sexual care or support received**					
	Yes	58 (65)		9 (45)	
	No	31 (35)		11 (55)	
**Timing of any previous sexual care or support received**					
	At diagnosis	16 (18)		5 (56)^a^	
	During treatment	10 (11)		4 (44)^a^	
	Both	32 (37)		0 (0)^a^	
**Type of prostate cancer treatment received**					
	Surgery only	53 (60)		17 (85)	
	Radiotherapy only	9 (10)		0 (0)	
	Hormone therapy only	7 (8)		0 (0)	
	Combined therapy	20 (22)		2 (10)	
**Treatment phase**					
	Pretreatment or on ongoing treatment	20 (23)		16 (80)	
	Less than 6 months of completing treatment	37 (42)		4 (20)	
	More than 6 months after completing treatment	32 (36)		0 (0)	
**In a relationship**					
	Yes	82 (91)		20 (100)	
	No	7 (6)		0 (0)	
**Usual partner gender**					
	Female	81 (91)		20 (100)	
	Male	8 (9)		0 (0)	

^a^n=9.

### Efficacy Data

The data are normally distributed. On the basis of the mean differences in pre- and postintervention (3 months) self-reported measures, a significant improvement was observed in total composite efficacy scores (*t*=9.570; *P=*.001), with a medium estimated effect size (Cohen *d*=0.577; Table 2). For individual survey items, significant improvements were seen in mean scores for (1) participants’ understanding of how to manage the impact of treatment (*t*=3.660; *P=*.001) and (2) participants’ perceptions of their ability to maintain a satisfying sex life despite cancer treatment (*t*=7.643; *P=*.001). No significant effects were found for participants’ mean current level of sexual satisfaction or level of comfort when discussing sexual issues with a partner or a health professional.

**Table 2:** Mean differences in pre- and postintervention (3 months) self-reported efficacy measures with estimated effect sizes^a^.

Individual statement	Baseline score, mean (SD)	Score at 3 months, mean (SD)	*t* test (*df*)	*P* value^b^ (2-tailed)	Effect size (Cohen *d*)	Effect size interpretation^c^
I currently have a satisfying sex life	1.89 (0.92)	1.90 (0.94)	0.376 (88)	.71	0.01	Insignificant
I have a good understanding of how to manage the impact of prostate cancer treatment on my sex life	2.31 (0.86)	2.57 (0.85)	3.660 (88)	.001^d^	0.517	Medium
I can have a satisfying sex life despite prostate cancer treatment	2.38 (0.79)	3.17 (0.78)	7.643 (88)	.001^d^	1.001	Large
I am comfortable discussing sexual issues with a partner	3.08 (0.72)	3.12 (0.7)	0.851 (88)	.40	0.055	Insignificant
I am comfortable discussing sexual issues with a health professional	2.81 (0.74)	2.82 (0.71)	0.241 (88)	.81	0.014	Insignificant

^a^Individual statements were scored on a scale between 1 and 4 points based on the response to the following: “How much do you agree with each statement?” Responses were measured on a 4-point scale anchored by *strongly disagree* and *strongly agree* at either end. Total composite scores out of 20 were calculated by combining scores from each statement. The total composite scores are as follows: mean baseline score, 12.23 (SD 2.46); mean score at 3 months, 13.62 (SD 2.31); t_88_=9.570; *P*=.001; effect size (Cohen *d*)=0.577; and effect size interpretation, medium.

^b^Bonferroni-adjusted *P* value for multiple comparisons (*P*=.007).

^c^Effect size interpretation: 0.00-0.19 (insignificant), 0.20-0.49 (small), 0.50-0.79 (medium), and ≥0.80 (large).

^d^Denotes a significant pre-post intervention effect.

### Intervention Use Data

**Figure 3:**
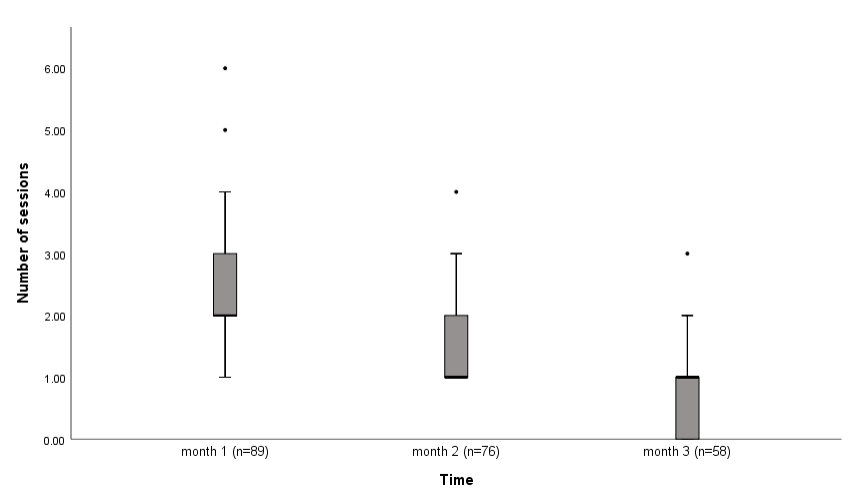
Box plot showing the number of sessions in each month over the duration of the intervention period.

**Figure 4:**
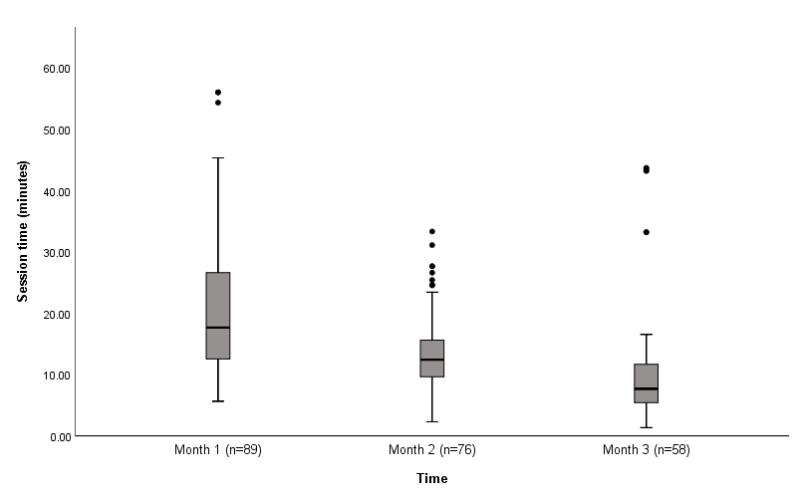
Box plot showing the duration of each session during the intervention period.

An analysis of program use during the 3-month intervention phase indicated that engagement with the intervention varied, suggesting that the participants used the intervention differently based on their individual needs and preferences. The participants completed a median of 3 sessions (range 1-11). The median session duration was 22 minutes (range 8-77), with an overall total use time of 78 minutes (range 18-284; Table 3). Of the 89 participants, 45 (51%) completed at least 4 of the 5 intervention steps and were subsequently classified as *completers*. Although the number of sessions and duration of each session reduced each month during the intervention period (Figure 3 and Figure 4), 85% (76/89) and 65% (58/89) of the participants were still using the intervention in the second and third months, respectively, of the intervention phase.

**Table 3:** Median and mean values for program use data.

Use measure	Value, median (IQR)	Value, mean (SD)
Number of sessions	3 (4)	3.8 (1.98)
Duration of each session (minutes)	22 (18)	36.4 (16.7)
Duration of total use time (minutes)	78 (80)	115.2 (43.5)

### Usability and Acceptability Data

On the basis of the postintervention survey data, the overall usability scores were found to be acceptable (total composite score: 19.68/24, 82% agreement). The highest levels of agreement were observed for the questions on *trust in the* *programme* (3.36/4, 93% agreement) and *information included was useful to me* (3.77/4, 94% agreement). The lowest agreement scores were found for the questions *I liked the look of the programme* (2.87/4, 72% agreement) and *I found the programme easy to use* (3.03/4, 76% agreement). Two-tailed, independent sample *t* tests identified that there were no significant differences in composite or individual question scores on the usability survey between the participants classified as *completers* and those classified as *noncompleters* (Table 4). Of the 89 participants, 70 (79%) agreed that they would recommend the intervention to others. The participants’ responses to questions on what they gained from using the intervention and any recommendations on how it could be improved are summarized in Table 5.

**Table 4:** Mean and percentage agreement scores for usability survey data (N=89)^a^.

Individual statement	Score, mean (SD)	Agreement (n=4), n (%)	Completers^b^ (n=45), mean score (SD)	Noncompleters (n=44), mean score (SD)	*P* value (difference between completers and noncompleters)
I was satisfied with the program	3.16 (0.56)	3.16 (79)	3.21 (0.68)	3.11 (0.71)	.44
I found the program easy to use	3.03 (0.69)	3.03 (76)	3.15 (0.7)	2.91 (0.67)	.10
I was able to move through the program easily	3.49 (0.56)	3.49 (87)	3.57 (0.56)	3.42 (0.61)	.37
I liked the look of the program	2.87 (0.47)	2.87 (72)	2.89 (0.67)	2.85 (0.71)	.45
I felt I could trust the program	3.36 (0.55)	3.36 (93)	3.26 (0.54)	3.47 (0.65)	.34
The information included in the program was useful to me	3.77 (0.53)	3.77 (94)	3.84 (0.55)	3.71 (0.48)	.63

^a^Individual statements were scored on a scale between 1 and 4 points based on the response to the following: “How much do you agree with each statement?” Responses were measured on a 4-point scale anchored by *strongly disagree* and *strongly agree* at either end. Total composite scores out of 24 were calculated by combining scores from each statement. The total composite scores were as follows: mean score, 19.68 (SD 0.56); agreement (19.68/24, 82%); mean completer score, 19.92 (SD 0.61); mean noncompleter score, 19.47 (SD 0.63); and *P*=.24.

^b^Completers were defined as participants who completed at least 4 of the 5 steps of the web-based program.

**Table 5:** Summary of participant comments on what they gained from using the intervention and recommendations on how it could be improved (N=207).

Explanation		Comments, n (%)	Category
**Views on what was gained from using intervention (n=141)**			
	The program provided information on issues not previously thought about	33 (23.4)	Information
	The program provided useful warnings on possible effects of treatment	26 (18.4)	Information
	The program helped to normalize sexual problems	22 (15.6)	Information and tone or language
	The program provided new information not previously discussed with health professionals	20 (14.2)	Information
	The program provided information that was relevant and useful to me as an individual	11 (7.8)	Information and personalization
	The program helped provide ideas for different approaches to manage sexual problems	9 (6.4)	Information and confidence or self-efficacy
	The program provided information that could be viewed and discussed with a partner	9 (6.4)	Information and communication
	The program provided a positive tone and message, which was reassuring	5 (3.5)	Tone, language and confidence, or self-efficacy
	The program helped to increase my confidence	4 (2.8)	Confidence or self-efficacy
	The program provided a reminder of information that was previously discussed with health professionals	2 (1.4)	Information
**Suggested improvements that could be made to the intervention (n=66)**			
	Make intervention available to patients before treatment starts	32 (48.5)	N/A^a^
	Make intervention available as a mobile app	17 (25.7)	N/A
	Include more support such as someone to contact for advice	14 (21.2)	N/A
	Make intervention available in an offline or printed format	3 (4.5)	N/A

**^a^**N/A: not applicable.

## Discussion

### Principal Findings

The findings from this study provide evidence of the efficacy of a web-based intervention designed to maximize sexual well-being in men living with prostate cancer. An analysis of self-reported outcome data found that the intervention resulted in significant improvements at the 3-month follow-up in overall efficacy scores and participants’ understanding of how to manage the impact of sexual concerns as well as their perceived ability to have a satisfying sex life despite prostate cancer treatment. The findings also indicated that the program had good overall usability and acceptability. Although the participants used the web-based self-management intervention in markedly different ways, they typically engaged well, taking part in multiple sessions during the intervention period. This is one of the first studies to evaluate the potential effectiveness and use of a tailored, sexual well-being support intervention for men living with prostate cancer and their partners, which is delivered using a web-based platform. A key strength of the intervention seems to be its flexibility with support that can be personalized based on the user’s needs and delivered at any stage of care.

### Intervention Efficacy

Self-reported measures at the 3-month follow-up were used to evaluate intervention efficacy, and they demonstrated significant overall improvements in comparison with the baseline scores. Improvements with a medium effect size were found in the extent to which the participants agreed that they had sufficient information to manage the impact of prostate cancer on their sex life. In addition, the extent to which the participants agreed that there was potential for them to have a satisfying sex life following treatment also improved significantly. The findings indicate that the intervention had a positive influence on the men’s self-perceived knowledge and understanding of how to manage sexual issues, but, more importantly, it also seemed to contribute toward a substantial increase in self-efficacy or a belief that a satisfactory sex life could be achieved following treatment. This indicates a potentially important prerequisite for maintaining behavioral change. Higher coping self-efficacy can result in more effective responses to behavioral barriers or setbacks, with individuals more able to apply behavior change maintenance strategies such as action planning [45,46].

An examination of the individual survey item scores suggested that the intervention had no effects on the participants’ current level of satisfaction with their sex life or on the level of comfort in discussing sexual issues with a partner or with a health care professional. The extent to which the participants agreed that they were happy with their current level of satisfaction was low at baseline, and although this may have been a factor for motivating potential participants to take part in the study, the intervention did not lead to improvements in this measure. This may have been due to the comparatively short timescale of the evaluation phase. Changes in sexual function after treatment are dependent on treatment type [9], with many effects having a long-term or persistent impact. Coping with these changes and adapting new practices as part of an individual’s sex life can take time. It may be necessary to use longitudinal studies to evaluate interventions aimed at improving satisfaction with current sex life, which is a complex, multifactorial concept that is closely related to the overall quality of life [47] and potentially mediating factors such as relationship status and expectations of recovery. The extent to which the participants agreed that they were comfortable when discussing sexual issues also did not change, but these scores were relatively high at baseline. This supports the findings from studies that have found that the level of comfort in men with prostate cancer is not a significant barrier to discussing sexual issues [21,48].

### Use, Usability, and Acceptability

Overall, the findings indicated that although the participants accessed the intervention a median of 3 times, the patterns of use seemed to differ among the participants. For example, engagement varied with some using the intervention more frequently over a number of shorter sessions throughout the intervention phase and others using it a limited number of times but with longer intervention sessions. This is reflected in the wide range of session numbers and session durations of between 1 and 11 sessions and 8 and 77 minutes, respectively. Although use reduced over the intervention period, approximately 65% (58/89) of the participants still showed engagement with the program in the final month. Previous evidence has demonstrated levels of engagement with web-based interventions that are comparable with face-to-face delivery methods [49], and it has been suggested that web-based resources are viewed as an acceptable and widely used source of information on sexual concerns [50]. Various needs of web-based interventions have been identified, including improving couple communication and providing information on sexual side effects, rehabilitation approaches, and realistic expectations of recovery [51]. The reasons for the variation in user engagement in this study may be related to a number of factors, including the fact that the intervention was intended to be used differently based on users’ individual needs and preferences. There is also evidence of an association between perceived usability or ease of use and engagement [52]. Usability and design issues could be additional reasons that may account for the different user engagement in this study. Although no significant differences were found among the users who completed at least 4 of the 5 intervention steps and those who did not, there was a slightly lower score in the noncompleter group in terms of their agreement with the question on the ease of using the program, and some participants may have discontinued use of the intervention because of technical or usability issues. Although overall the intervention was seen as acceptable to the participants with a good level of engagement observed, it is critical that any usability or acceptability issues are explored in detail and addressed in future redesigns of the intervention. This is important to maximize engagement because the intention to use web-based interventions is mediated by perceived ease of use, usefulness, and social determinants. Increased engagement may be related to behavioral or demographic characteristics, including previous experience of using web-based programs [40,53,54]. In addition, the mode of intervention delivery may have had an influence on engagement, particularly in terms of the number of recorded sessions. The intervention was designed for use on a laptop or desktop computer. These may be accessed less frequently than mobile devices; therefore, delivery of the intervention in a mobile app form might have increased the number of sessions completed by the participants. This was reflected in some of the views on the intervention, with availability of an app format being the second highest suggested improvement that could be made (Table 5).

Use may also have been affected by the behavioral components of the intervention. Although the intervention included common behavior change techniques such as information on health consequences, social support, and use of reminders and prompts, it did not include other methods commonly associated with sustained and repeated use of web-based programs, for example, regular self-monitoring or the use of goal setting. Although the flexibility and open access of the intervention (ie, not locking steps until completion of a previous step) may have increased initial engagement, it might also have been anticipated that this might make it less likely that the participants would return to the intervention as frequently. However, this was not the case, and a reason for this may have been that the users returned to review previous information. This is evidenced by data from the usability survey, which indicated that overall, the intervention was seen as being usable, with the tailored information provided regarded as useful and relevant (Table 4). Another key reported benefit of the intervention was that the participants reported a high level of trust in the information provided and reported that it helped to facilitate them and their partners to initiate conversations about sexual well-being that they might not otherwise have had (Table 4). This was observed despite the participants reporting relatively high levels of comfort in discussing sexual issues with a partner (Table 2). Couple communication about sexual well-being can be regarded as complex and often difficult to initiate [21]. However, such communication is important and is an essential step in managing concerns and supporting sexual well-being recovery.

### Limitations

One limitation is that we were unable to explore in detail the reasons for the withdrawal of some participants from the study or examine the factors responsible for increased engagement with the intervention. The sample of participants was also relatively homogeneous, which may limit the generalizability of the study findings. The assessment of usability was based on a modified version of the system usability scale [43], which limits the ability to compare the findings on program usability with those of other studies.

### Conclusions

In this paper, efficacy, use, and acceptability data are presented for a tailored web-based intervention designed to maximize sexual well-being in men living with prostate cancer. This study provides preliminary evidence for the efficacy of the intervention, which was perceived as being usable and acceptable to the participants with evidence of sustained use. Digital interventions may provide access to low-cost, scalable, updatable, and evidence-based information for managing sexual concerns after prostate cancer treatment. By acknowledging the impact of treatment on sexual well-being and providing appropriate support at all stages of care, this intervention might have the potential to improve patient-important outcomes and could easily be made available in routine practice. Further research will be conducted to explore the factors associated with increased engagement, and these findings will be used to refine content before testing as part of larger longitudinal and randomized controlled studies examining longer-term intervention effectiveness on a wider range of patient-important outcomes, including symptom distress, self-efficacy, knowledge, couple communication, sexual satisfaction, and overall quality of life. These studies will also be used to examine the influence of key demographic factors such as age profile, treatment type, relationship status, and sexual orientation on these outcomes.
